# Sequencing the Genome of Indian Flying Fox, Natural Reservoir of Nipah Virus, Using Hybrid Assembly and Conservative Secondary Scaffolding

**DOI:** 10.3389/fmicb.2020.01807

**Published:** 2020-07-29

**Authors:** Julien Fouret, Frédéric G. Brunet, Martin Binet, Noémie Aurine, Francois Enchéry, Séverine Croze, Marie Guinier, Abdelghafar Goumaidi, Doris Preininger, Jean-Nicolas Volff, Marc Bailly-Bechet, Joël Lachuer, Branka Horvat, Catherine Legras-Lachuer

**Affiliations:** ^1^CIRI, International Center for Infectiology Research, Team Immunobiology of Viral Infections, Univ Lyon, INSERM U1111, CNRS UMR 5308, Ecole Normale Supérieure de Lyon, Université Claude Bernard Lyon 1, Lyon, France; ^2^Viroscan3D, Trévoux, France; ^3^Institut de Génomique Fonctionnelle de Lyon, Université de Lyon, CNRS UMR 5242, Ecole Normale Supérieure de Lyon, Université Claude Bernard Lyon 1, Lyon, France; ^4^Plateforme Profilexpert, Université Claude Bernard Lyon 1, Lyon, France; ^5^Tiergarten Schönbrunn, Vienna, Austria; ^6^Université Nice Sophia Antipolis, INRA, CNRS, ISA, Nice, France; ^7^Cancer Research Center of Lyon, INSERM 1052/CNRS 5286, Université de Lyon, Lyon, France; ^8^Ecologie Microbienne, CNRS UMR 5557, LEM, INRA, VetAgro Sup, Université Claude Bernard Lyon 1, Villeurbanne, France

**Keywords:** hybrid genome assembly, Nipah virus, *Pteropus medius*, *Pteropus giganteus*, secondary scaffolding, bats

## Abstract

Indian fruit bats, flying fox *Pteropus medius* was identified as an asymptomatic natural host of recently emerged Nipah virus, which is known to induce a severe infectious disease in humans. The absence of *P. medius* genome sequence presents an important obstacle for further studies of virus–host interactions and better understanding of mechanisms of zoonotic viral emergence. Generation of the high-quality genome sequence is often linked to a considerable effort associated to elevated costs. Although secondary scaffolding methods have reduced sequencing expenses, they imply the development of new tools for the integration of different data sources to achieve more reliable sequencing results. We initially sequenced the *P. medius* genome using the combination of Illumina paired-end and Nanopore sequencing, with a depth of 57.4x and 6.1x, respectively. Then, we introduced the novel scaff2link software to integrate multiple sources of information for secondary scaffolding, allowing to remove the association with discordant information among two sources. Different quality metrics were next produced to validate the benefits from secondary scaffolding. The *P. medius* genome, assembled by this method, has a length of 1,985 Mb and consists of 33,613 contigs and 16,113 scaffolds with an NG50 of 19 Mb. At least 22.5% of the assembled sequences is covered by interspersed repeats already described in other species and 19,823 coding genes are annotated. Phylogenetic analysis demonstrated the clustering of *P. medius* genome with two other *Pteropus* bat species, *P. alecto* and *P. vampyrus*, for which genome sequences are currently available. SARS-CoV entry receptor ACE2 sequence of *P. medius* was 82.7% identical with ACE2 of *Rhinolophus sinicus* bats, thought to be the natural host of SARS-CoV. Altogether, our results confirm that a lower depth of sequencing is enough to obtain a valuable genome sequence, using secondary scaffolding approaches and demonstrate the benefits of the scaff2link application. The genome sequence is now available to the scientific community to (i) proceed with further genomic analysis of *P. medius*, (ii) to characterize the underlying mechanism allowing Nipah virus maintenance and perpetuation in its bat host, and (iii) to monitor their evolutionary pathways toward a better understanding of bats’ ability to control viral infections.

## Introduction

Bats have been reported to be the natural reservoir of several zoonotic viruses that cause severe human diseases, including Marburg, Ebola, Nipah, Hendra, SARS, and MERS viruses (Wang and Anderson, 2019). *Pteropus medius* (also known as *P. giganteus* and commonly called Indian flying fox (Mlikovsky, 2012) is a frugivorous giant bat, widely distributed in Southeast Asia, and shown to host numerous viral species (Anthony et al., 2013), including Nipah virus (Yadav et al., 2019). Nipah virus is a recently emerged zoonotic *Paramyxovirus*, capturing the attention of both scientific and public health communities due to its high lethality rate, up to 90% in Bangladesh and India epidemics, associated with human-to-human transmission (Mathieu and Horvat, 2015; Arunkumar et al., 2019). Although this virus is highly pathogenic in humans and numerous other mammalian species, it is asymptomatic in its natural host, fruit bats (Enchéry and Horvat, 2017). A better understanding of virus–host interactions requires further studies necessitating the availability of the sequenced genome (Shi, 2010), which has been lacking for *P. medius*. Therefore, our work aims to provide the genome sequence of *P. medius* as a new resource for genomic studies, analysis of molecular basis of bats’ unique adaptation and bats’ immunovirology and should help in understanding the underlying evolutionary mechanisms used by emerging viruses, like Nipah virus, to cross species barriers and to widespread in the newly introduced host. Moreover, recent outbreaks of numerous pathogenic viruses from bats, including SARS coronavirus (Hu et al., 2015), urge improved comprehension and characterizations of bat species.

Although bats make up more than 20% of existing mammals with 1,400 species (Lazzeroni et al., 2018), only 38 bat genome assemblies are currently available in the NCBI Genbank database. Several different sequencing strategies have been used in the past years to sequence bat genomes, including (i) high coverage long-read sequencing strategy, e.g., 83x of PacBIO *Eonycteris spelaea* (Wen et al., 2018), (ii) Illumina sequencing (e.g., 93x for paired-end libraries and 67x of mate-pair libraries) for *P. alecto* (Zhang et al., 2013) and also (iii) hybrid sequencing, e.g., 145x of Illumina short reads and 24x of PacBio long-reads for *Rousettus aegyptecus* (Pavlovich et al., 2018). Combined technological developments applied to genome assembly, including Hi-C (Ghurye et al., 2018), optical mapping (Jiao et al., 2017) and synthetic long reads (Coombe et al., 2016) provide a possibility for the high quality chromosome-level assembly (Teeling et al., 2018). In contrast to direct sources of evidence used for scaffolding the genome sequence, secondary scaffolding leverages less direct type of information to improve assembly scaffolding. In this report, we focus on a reference-assisted assembly method using Ragout (Kolmogorov et al., 2014) and a gene-based method using AGOUTI (Zhang et al., 2015) that require a reference genome and RNA-Seq data, respectively (Zhang et al., 2013; Pavlovich et al., 2018; Wen et al., 2018).

We used a combination of Illumina paired-end reads and Nanopore long-reads sequencing with coverage of 57.4x and 6.1x, respectively, based on a genome size estimation of 2 Gb. In addition, the sequencing results in this study have benefited from the produced RNA-seq data. Although the sequencing coverage obtained in this study was lower than in some other recent reports, we show here that it has been enough to provide a good quality genome using current computational method. In the first step, we provide an assembly based directly on sequencing evidence (paired-end and long-reads), where assembled fragments are called solid scaffolds. Then, we improve the assembly using secondary scaffolding methods, AGOUTI (gene-based) and Ragout (reference-assisted), using RNA seq data obtained with an Illumina 75 bp paired-end sequencing. Finally, we provide an original tool, called scaff2link, which integrates both sources of secondary scaffolding to keep only non-discordant linkages, hence avoiding potential errors. The development of these methods is essential for a better use of available resources, particularly when the number of the high-quality genome sequences is expected to increase rapidly. To follow the impact of the different methods regarding the quality of the assembly, we next describe metrics for each mentioned step: solid scaffolds, secondary scaffolding and the use of scaff2link. Therefore, in the same order, the metric described in this study contains: (i) fragmentation state of the genome sequence; (ii) benchmark using single-copy orthologs (SCO), and (iii) DNA-Seq realignment consistency. To characterize the nature of the sequence provided as a resource, we performed a k-mer analysis on solid scaffolds, describing the state of ploidy. Based on quality metrics, we show that the best assembly was obtained after integration of both secondary scaffolding methods with scaff2link. Finally, we demonstrate the potential use of the obtained genome assembly by providing annotation of both coding genes and interspersed repeats and performing the phylogenetic analysis.

This new approach allowed us to assemble the genome of *P. medius* with a length of 1,985 Mb, consisting of 33,613 contigs and 16,113 scaffolds with a NG50 of 19 Mb. Identified repeat elements covered 22.5% of the assembled sequences and 19,823 coding genes were annotated, thus providing the first genome sequence of this bat species and making it available for further studies and better understanding of the mechanisms of Nipah virus emergence.

## Materials and Methods

### *Pteropus medius* Sampling and Cell Generation

**FIGURE 1:**
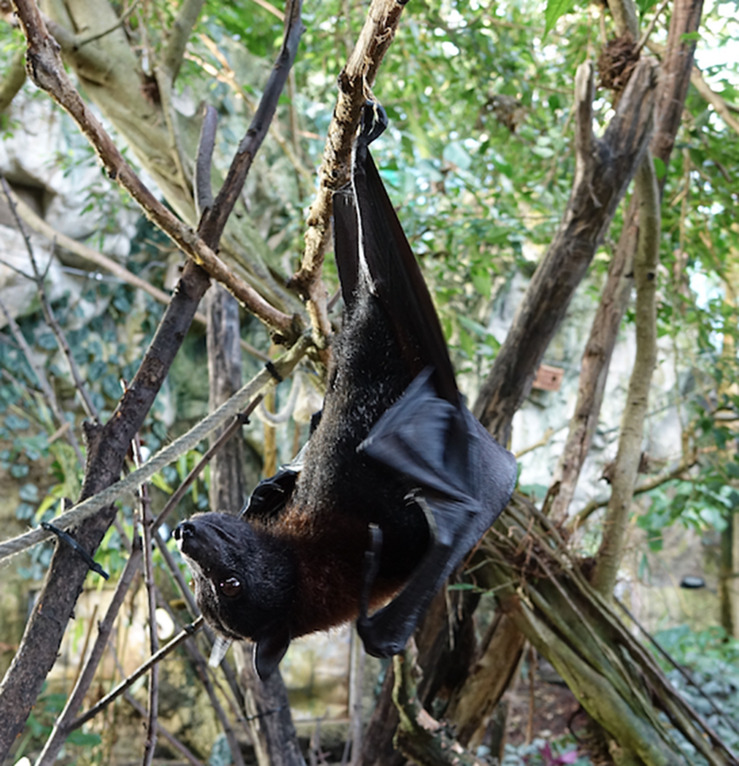
Fruit bat *Pteropus medius*. Female specimen of *P. medius*, called Daya, born in 24 March, 2009 in the Tiergarten Schönbrunn, Vienna, Austria. Its wing-membrane skin biopsy was used for the generation of primary cell line and subsequent genome sequencing.

Cell culture from *Pteropus* bat flying fox was generated from a wing-membrane skin biopsy of a female specimen of *P. medius* (known also as Indian flying fox and *P. giganteus*, belonging to *Yinpterochiroptera* suborder) (Figure 1), collected in the Tiergarten Schönbrunn (Vienna, Austria), during the regularly veterinary check, as previously described (Gaudino et al., 2020). Briefly, sample was washed with sterile PBS and transferred into freezing medium Cryo-SFM (PromoCell Bioscience) in dry ice, for the shipment from the zoo. To obtain a primary cell culture (Ptgv), samples were thawed and fractioned in a Petri dish, then homogenates were harvested in different media and cultured in DMEM/F-12 medium (Gibco) supplemented with 10% fetal bovine serum, 1% L-glutamine (200 mM), 1,000 U/mL of penicillin, 1,000 U/mL of streptomycin, at 37°C, at 5% CO2. We confirmed that the biological sample belongs to the *P. medius* species by sequencing a mitochondrial region D-loop (Saccone et al., 1987) and nuclear introns ACOX2, COPS7A, BGN, ROGD1, and STAT5A, suggested to be pertinent to distinguish among closely related species (Dool et al., 2016). We compared these sequences with those obtained from several individuals phenotypically identified as *P. medius*, sampled at National Institute of High Security Animal Diseases, Bhopal, India.

### Illumina DNA-Seq

Genomic DNA was purified from a pelleted primary Ptgv cells using NucleoSpin^®^ Tissue kit (MACHEREY-NAGEL, 740952). A paired-end library was constructed with an insert size of 462 bp in average using NEXTFLEX^®^ PCR-Free DNA-Seq Kit (Bioo Scientific) with Bioruptor (Diagenode) for DNA fragmentation step. Paired-end short-reads of 150 bp were generated on Illumina NextSeq 500 platform using a high output flowcell.

### Illumina RNA-Seq

Primary cell cultures of Ptgv cells, containing principally fibroblast-like cells, were used as a source of RNA. Three replicates were obtained for each condition 24 h post-infection, either from Nipah virus-infected Ptgv cells at three viral particles/cell or from non-infected cells (incubated with mock preparation). The level of Nipah virus infection was bellow 3% in Ptgv cells. Experiments were done in BSL-4 facility INSERM Jean Mérieux in Lyon. Supplementary samples were obtained from another *Pteropus* bat trachea tissue (Aurine et al., 2019). RNA was extracted using NucleoSpin^®^ RNA (MACHEREY-NAGEL, 740955). Libraries were generated using NEXTFLEX^®^ Rapid Directional RNA-Seq Library Prep Kit according to manufacturer’s protocol (Bioo Scientific) using 185 ng of extracted RNA as input material. Libraries were sequenced via Illumina NextSeq 500 in paired-end 75 bp using a Mid-Output Flow Cell.

### DNA Extraction for MinION Sequencing

High-molecular-weight DNA was extracted from cell culture using the MagAttract^®^ HMW DNA Kit (Qiagen). Then, genomic DNA was sheared using Covaris g-TUBE as per manufacturer’s guidelines to obtain approximately 10 kb fragments without any additional enrichment or purification step.

### Quantity and Quality Measurement for DNA MinION Sequencing

DNA concentration was measured using the Quantus^TM^ Fluorimeter with the QuantiFluor^®^ dsDNA Kit (Promega), and purity was evaluated by measuring A260/A280 and A260/A230 ratios using a NanoDrop^TM^ spectrophotometer. The quality of DNA and its size were assessed by electrophoretic migration using Fragment Analyzer with the High Sensitivity Large Fragment 50 Kb Analysis Kit (AATI).

### Library Preparation for MinION Sequencing

Sequencing libraries were prepared using the Ligation Sequencing Kit 1D (SQK-LSK108) or the 1D^2^ Sequencing Kit (SQK-LSK308) (Oxford Nanopore Technologies). The libraries were then loaded to MinION using a flow cell R9.5 version (FLO-MIN106) (ONT) and the sequencing runs were performed under MinKNOW version 1.7.14 for up to 48 h.

### Illumina Primary Analysis

Raw reads were trimmed using cutadapt (version 1.9.1 with options: -q 30 -m 115 for DNA-seq and -q 30 -m 50 for RNA-Seq). For DNA-seq, resulting reads were corrected using lighter (version 1.1.1). Expected genome size has been predicted using kmergenie software (v1.7016).

### MinION Primary Analysis

Base calling was done through albacore (v2.1.10). Low quality ends and chimera reads were trimmed using porechop (v0.2.3). Resulting long-reads were filtered using filtlong with short DNA-Seq Illumina reads to estimate quality (v0.2.0 with options: –trim –split 250 –min_length 200 –min_mean_q 85, –length_weight 0 and –window_q_weight 0).

### Assembly and Primary Scaffolding

Hybrid assembly using both Illumina short-reads and Nanopore long-reads was achieved using MaSurCA whole genome assembly software (v3.2.4) (Zimin et al., 2013), with a k-mer size of 97. This value was chosen after preliminary tests (described in the Supplementary Material). Gap filling was performed on this assembly using gapfiller (v1.10) with 20 iterations.

### Secondary Scaffolding

For gene-based scaffolding, RNA-Seq data has been aligned using *hisat2* (v2.1.0) on the assembly. Gene annotation have been predicted using *augustus* (v3.3.1) with human training set. Finally, these data were integrated with AGOUTI (v0.3.2).

For reference-based scaffolding, genome sequences of *P. vampyrus* (pteVam2), *P. alecto* (pteAle1), and *R. aegyptecus* (Raegyp2) were extracted from Genbank database and aligned with the solid-scaffold assembly using progressive Cactus (https://github.com/glennhickey/progressiveCactus). The software Ragout v2.1 performed the scaffolding based on the multi genome alignment using both pteVam2 and pteAle1 assembly sequences as reference, Raegyp2 as outgroup and the solid scaffold assembly as target (Kolmogorov et al., 2018).

### Scaff2link Script

We developed an algorithm to integrate, in a conservative way, scaffolding results given by Ragout and AGOUTI (available at: https://github.com/jfouret/scaff2links). Briefly, this algorithm builds a graph with phylogenetic and gene-based linkage information as input for edges, and fasta formatted sequences as input for nodes (Supplementary Figure S1). First, linear paths are simplified and then higher level of simplification is added using pattern of directed acyclic graph (DAG) (Supplementary Figure S2). The pseudocode of the algorithm is given in Supplementary Material.

### Assembly Evaluation

NG50 have been computed for all assemblies using a genome size of 2 Gb. K-mer Analysis Toolkit (v2.3.1) have been used to compare 23-mers presence and multiplicity in Illumina reads and in assembly. BUSCO (v3) has been used to control the presence in single copy of SCO shared in *Laurasiatheria* super order. Illumina DNA-Seq reads were mapped with bowtie2 (v 2.2.9) with options to consider an alignment properly paired if fragment size is in range 150 (-I) and 2,000 (-X) and “-very-sensitive” mode. More specifically, metrics were computed using “flagstat” tools of the SAMtools suite (v1.9).

### Genome Annotation

Genomes from *P. vampyrus* (pteVam2), *P. alecto* (pteAle1), and *R. aegyptecus* (Raegyp2) and the Ma_sr-lr_union100 assembly were aligned using progressive Cactus (https://github.com/glennhickey/progressiveCactus) (Paten et al., 2011). *R. aegyptecus* was used as outgroup to improve the multiple alignment process and has been chosen because it was a species close to the *Pteropus* genus with a good quality assembly. Gene annotation was performed using the Comparative Annotation Toolkit (CAT) v0.1 (Zhang et al., 2013) automated pipeline with *P. alecto* as reference and *P. medius* as target with a step of *ab initio* gene prediction using Augustus 3.3.1 (Stanke and Waack, 2003). Of note, *R. aegyptecus* genome assembly has been used solely to improve the multiple genome alignment of the three *Pteropus* species genomes, however, *R. aegyptecus* was not used directly to infer *P. medius* genome annotation. CAT is using other dependencies: Hal v1, SAMtools 1.7–2 and BEDtools v2.26.0. Repeated sequences were annotated with *RepeatMasker* (v open-4.0.7) using rmblast (Nucleotide-Nucleotide BLAST with *RepeatMasker* Extensions 2.2.27+) and RepeatMasker Combined Database (Dfam_Consensus-20170127, RepBase-20170127) filtered for mammal species. Additional option for *RepeatMasker* were “-s -nolow -no_is.”

### Phylogenetic Analysis

Proteomes were retrieved from NCBI for *Miniopterus natalensis* (GCF_001595765.1), *Eptesicus fuscus* (GCF_000308155.1), *Myotis brandtii* (GCF_000412655.1), *M. lucifugus* (GCF_000147115.1), *M. davidii* (GCF_000327345.1), *Rousettus. aegyptiacus* (GCF_001466805.2), *P. alecto* (GCF_000325575.1), *P. vampyrus* (GCF_000151845.1), *Rhinolophus ferrumequinum* (GCF_004115265.1), *Desmodus rotundus* (GCF_002940915.1), *Hipposideros armiger* GCF_001890085.1), and *Phyllostomus discolor* (GCF_004126475.1). More specifically “GCF_^∗^_ translated_cds.faa.gz” file was fetched from NCBI RefSeq FTP repository for each species. For *P. medius*, the proteome was translated from the annotation. In case of isoforms, only the longest isoform per gene was kept. SCO were identified thanks to OrthoFinder^1^ (Emms and Kelly, 2019) using default parameters. Multiple sequences alignment was conducted separately on each locus using MAFFT v7.123b (Katoh, 2002) with, generalized affine gap score, BLOSSUM80 (–bl) matrix for scores, an open gap penalty of 10 (–op 10) and all other parameters set to default. All positions with at least one gap were removed from the MSA along with surrounding five residues, as gaps may be linked with a missing or incorrect sequence. Of note, the models implemented in the phylogenetic tools used hereafter do not consider gaps in a multiple sequence alignment. Then conserved blocks were extracted from each MSA using Gblocks 0.91b (−b1 = 13, −b2 = 13, −b3 = 2, −b4 = 10) in order to improve the phylogeny (Talavera and Castresana, 2007). MSA were then concatenated. Maximum of 20 likelihood searches have been conducted with respectively 20 distinct starting trees to identify the best tree using RAxML 8.2.9 (Stamatakis, 2014). Separately, bootstrapping was done with 100 iterations. The random seed “12345” was used at all steps with RAxML. The tree was rooted manually. A MSA subset with 500,000 sites was used for estimation of divergence times using soft fossil constraints with PAML v4.9j mcmctree program (Yang, 2007). Configuration files used in PAML are available in the Supplementary Material. The procedure has been repeated with another subset with no significant changes in age time and confidence intervals. The minimum age of fossil data, obtained from paleobiodb database^2^, were used as lower bound; all values used for the calibration were specified (Supplementary Figure S3). An upper constraint was set for the root using the upper value of the 95% confidence interval available for the *Chiroptera* order on Time Tree database^3^ (Kumar et al., 2017).

## Results

### Sequencing Depth

*Pteropus medius* DNA was extracted from cultured primary bat cells and sequenced using both paired-end Illumina and Nanopore sequencing, generating 114.5 Gb of Illumina DNA-Seq data (after primary analysis; see section “Materials and Methods”). Based on the analysis on distinct k-mer counts in DNA-Seq short-reads, the software kmergenie (Chikhi and Medvedev, 2014) predicted a genome size from 1.92 to 1.98 Gb, corresponding to the range of size of previously published bat genomes (Teeling et al., 2018). Using an alternative method based on the C-value (Smith and Gregory, 2009), the genome size was estimated at 1,994 Gb, with a GC content of about 38% deduced from DNA-Seq data. We then used a genome size of 2 Gb to calculate the depth of sequencing and subsequently normalized assembly metrics. The sequencing depth obtained for Illumina paired-end data was 57.4x. For long-read data, 12.1 Gb of MinION were produced after primary analysis with a mean long-read size of 7 kb, corresponding to a depth of 6.1x. These data altogether were then used for the final assembly. We also isolated the cellular RNA and performed the RNA-Seq analysis using the same biological material, then used the produced data in the secondary scaffolding step.

### Solid Scaffold Assembly With Pieces of Evidence Directly Linked to Sequencing

**FIGURE 2:**
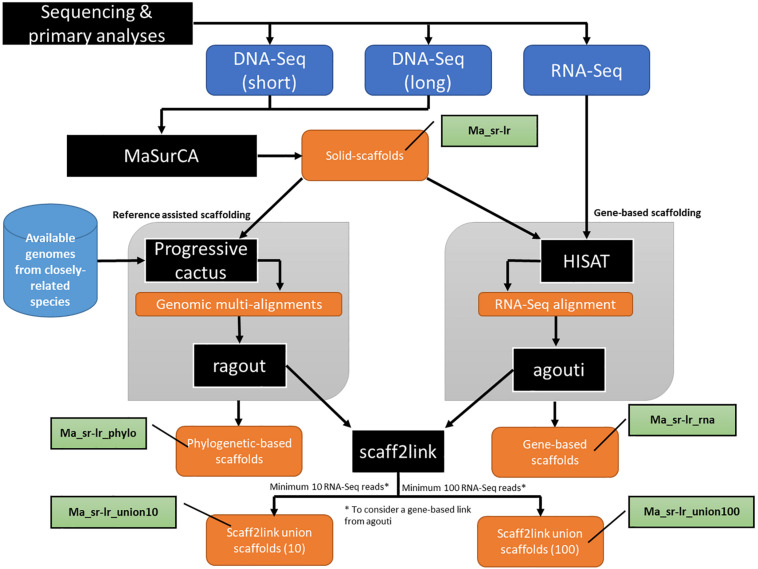
Schematic presentation of the main analytical steps of the assembly workflow of *Pteropus medius* genome. Analytic steps, including the applied software (MaSurCa, HISAT, Progressive cactus AGOUTI and Scaff2link) are marked in black, input data in dark blue, data obtained from databases in light blue, nomenclature used for produced assemblies in green and produced data in orange.

**FIGURE 3:**
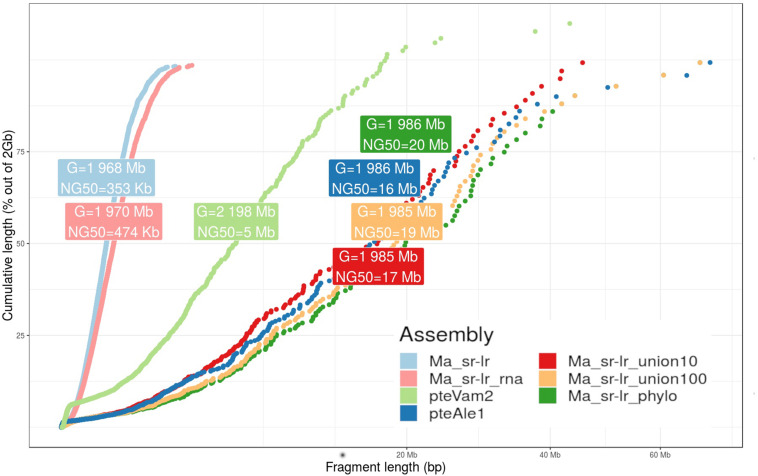
Fragmentation graph for produced assemblies. The graph presents a scatter plot comparing cumulative length (*y*-axis) and the fragment length (*x*-axis) for four different *P. medius* scaffold assemblies, compared with already published *P. vampyrus* (pteVam2) and *P. alecto* (pteAleI). For each assembly, cumulative lengths were computed along sorted fragments, from the smallest to the highest. A label box gives information about the total size of the assembly (G) and the NG50 values, where the genome size has been set to 2 Gb for normalization.

**FIGURE 4:**
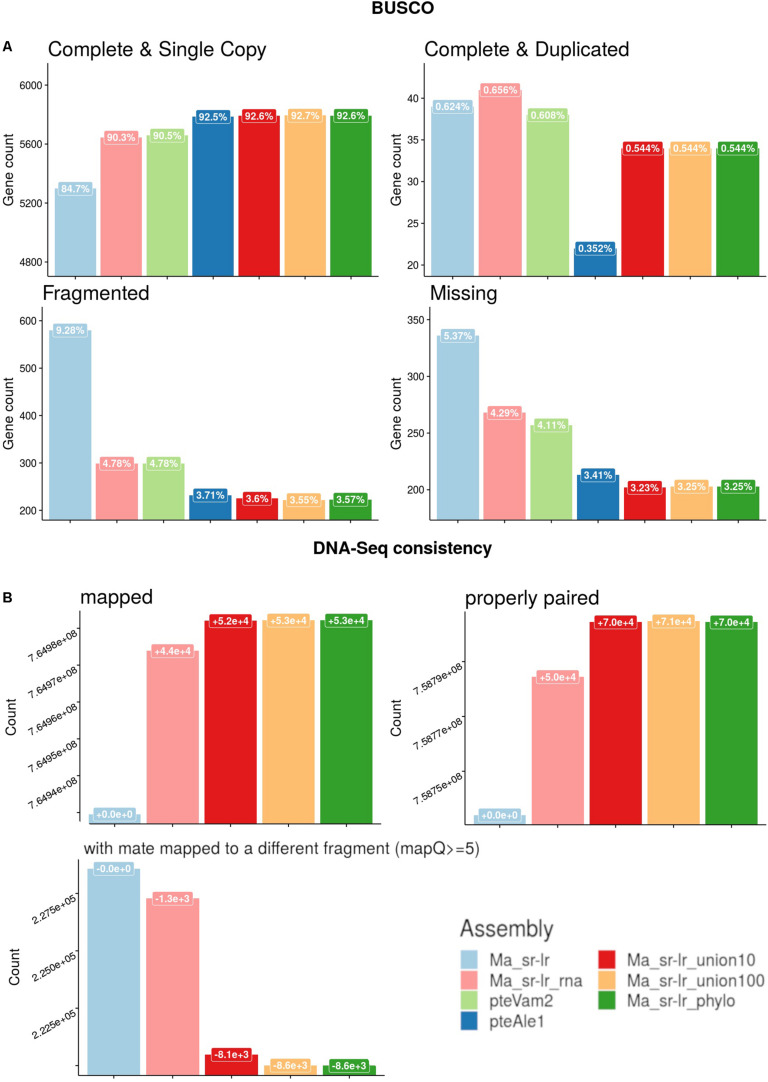
Quality assessments of produced assemblies. **(A)** Benchmark of universal single-copy orthologs (BUSCO) genes for the *Laurasitharia* super order with comparison to already published assemblies of two *Pteropus* species, *P. vampyrus* (pteVam2), and *P. alecto* (pteAleI). **(B)** Short-reads mapping against produced assemblies were analyzed. *Y*-axis is the number of reads that are either (i) mapped (ii) properly paired or (iii) mapped with quality superior to five and with a mate mapped to a different fragment. The label box on top of each bar chart presents the evolution of read counts compared to the solid scaffold assembly (Ma_sr-lr).

Both Illumina paired-end and MinION long-reads were assembled with the MaSurCA (Zimin et al., 2013) hybrid software and gaps were filled with gapfiller (see section “Materials and Methods”). The workflow of this step is part of the general workflow presented in Figure 2, where the assembly produced is named “Ma_sr-lr” for (Ma: MaSurCa, sr: short-reads, lr: long-reads). Metrics describing this assembly are the fragmentation state of the genome sequence (Figure 3), benchmark using SCO (Figure 4A) and DNA-Seq realignment consistency (Figure 4B).

Previously published *Pteropus* bat genomes from *P. vampyrus* (Lindblad-Toh et al., 2011) and *P. alecto* (Zhang et al., 2013) were used for comparative analysis. For the fragmentation presented on Figure 3, the Ma_sr-lr reached an NG50 of 353 kb, with a total length of 1.968 Gb. Ma_sr-lr NG50 metrics was ∼14 and ∼45 times lower compared to *P. vampyrus* (NG50: 5 Mb) and *P. alecto* (NG50: 16 Mb) assemblies, respectively (Figure 3). Focusing on the potential use of the genomes for gene annotation, we have used BUSCO (Benchmark of Universal Single Copy Orthologs). This approach attempts a homology-based annotation of a group of genes selected because their orthologs are present in at least 90% of species from *Laurasiatheria* in single copy. For “Ma_sr-lr,” only 84.7% of BUSCOs (Figure 4A) have been successfully annotated and confirmed to be present as a single copy, even if it has been lower than in *P. vampyrus* (90.5%) or *P. alecto* (92.5%). The relative difference with other *Pteropus* is lower than the fragmentation of the genomes, indicating that we might have reached a limit of genome contiguity which facilitates gene annotation. Indeed, 353 kb (NG50 of Ma_sr-lr) is in order of magnitude ∼35 times higher than the median gene size among species of the *Pteropus* genus; 11.5 kb for *P. vampyrus* (RefSeq 101) and 10.4 kb for *P. alecto* (RefSeq 102) (NCBI Resource Coordinators, 2017).

### k-mers Analysis Describing the State of Ploidy of the Solid Scaffold Assembly

**FIGURE 5:**
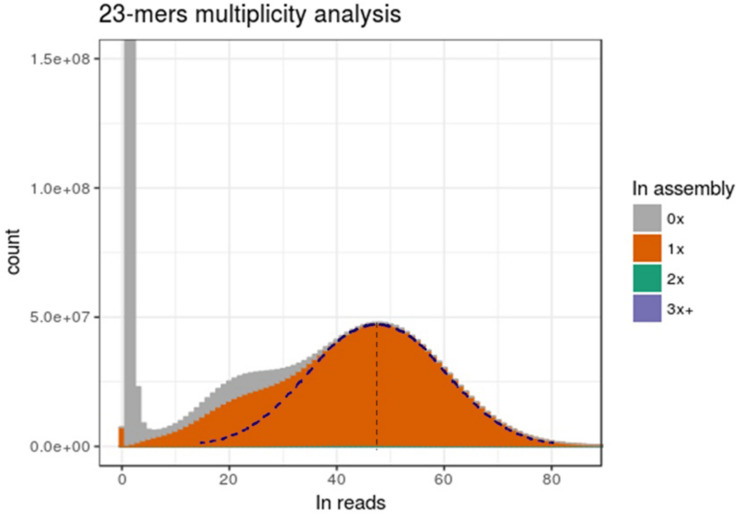
Genome assembly is representative of only one allele. The *x*-axis presents the multiplicity of a k-mers. The *y*-axis is the amount of distinct k-mers present at this multiplicity in reads. Fill colors represent the number of time (0x, 1x, 2x or 3x, and more) a distinct k-mer is present in assembly. The right part of the dotted Gaussian curve has been hand-traced and a symmetry has been applied to get the left part.

We then focused on comparing the distribution of k-mer multiplicity between DNA-Seq reads and the assembly. As shown in Figure 5, large number of k-mers with low multiplicity in Illumina DNA-Seq reads were not present in the assembly (corresponding to the first left peak, close to 0x); this is most probably an artifact from sequencing errors. This peak is followed by a bimodal Gaussian-like distribution, suggesting that those two mixed distributions correspond to the heterozygous k-mers (left curb; mean: ∼24x) and for homozygous k-mers (right curb; mean: ∼48x); we indeed expected homozygous k-mers to be twice more present in sequencing reads. Half of heterozygous k-mers were apparently not integrated in the assembly, while the remaining part was integrated only once (Figure 5). This suggests that most heterozygous positions were inserted only once in the genome assembly. On the other side, if two alleles were integrated in the assembly for a consequent number of polymorphic positions, we would expect to see a significant proportion of homozygous k-mers present at a multiplicity of two in the assembly; however, only 1.7% of the total k-mers is found twice in the assembly (not visible in the graph). Therefore, the assembly presented in this report is very likely to be representative of a haplotype.

### Secondary Scaffolding Using Reference-Assisted or Gene-Based Methods

The workflow used to perform secondary scaffolding is presented in Figure 2. Both methods start with the Ma_sr-lr assembly. The assembly produced after reference-assisted scaffolding is called “Ma_sr-lr_phylo.” The number of scaffolds (Supplementary Table S1), 29,459 for Ma_sr-lr, was diminished by 2,018 and 13,011 by gene-based scaffolding (Ma_sr-lr_rna) and reference-assisted (Ma_sr-lr_phylo) scaffolding, respectively. Quality was assessed based on different metrics as explained above.

The state of fragmentation of obtained assemblies is presented on Figure 3. While reference-assisted scaffolding showed a very high NG50 value of 20 Mb, the value for gene-based scaffolding was limited to 474 Kb (Figure 3). Interestingly, the value of NG50 for Ma_sr-lr_phylo assembly outperforms the NG50 of *P. alecto* assembly (16 Mb) (Zhang et al., 2013), while having a smaller number of Ns (any nucleotide according to the IUPAC code) in its sequence (Supplementary Table S1). During the scaffolding processes, Ns are produced when resolving a gapped linkage of two fragments.

Annotation metrics of Benchmark of Universal Single-Copy Orthologs (BUSCOs), is presented on the Figure 4A. The percentage of complete and single copy BUSCOs annotated raised from 84.7 to 90.3% (+5.6%) and 92.6% (+7.9%) for Ma_sr-lr_rna and Ma_sr-lr_phylo, respectively. It appears that in most cases this difference corresponds to fragmented BUSCOs in the solid-scaffold assembly, which can be annotated on a single fragment in Ma_sr-lr_rna and Ma_sr-lr_phylo assemblies (Figure 4A).

Finally, Figure 4B presents metrics of DNA-Seq Illumina read realignment. There is a slight increase in mapped reads for both Ma_sr-lr_rna and Ma_sr-lr_phylo assemblies, + 4.4⋅10^4^ and+5.3⋅10^4^ mapped reads (out of 7.65⋅10^8^), respectively. An increase within the same order of magnitude co-occurs for the number of properly paired reads. Given options to the aligners define proper alignment with the constraint of the fragment size to be between 500 and 2,000, as expected from sequencing. Finally, the number of reads with a mate mapped to different fragments had decreased. All these data together indicate that the most added linkages are consistent with DNA-Seq realignment.

### Scaff2links to Integrate Two Types of Linkage Source for Scaffolding

**FIGURE 6:**
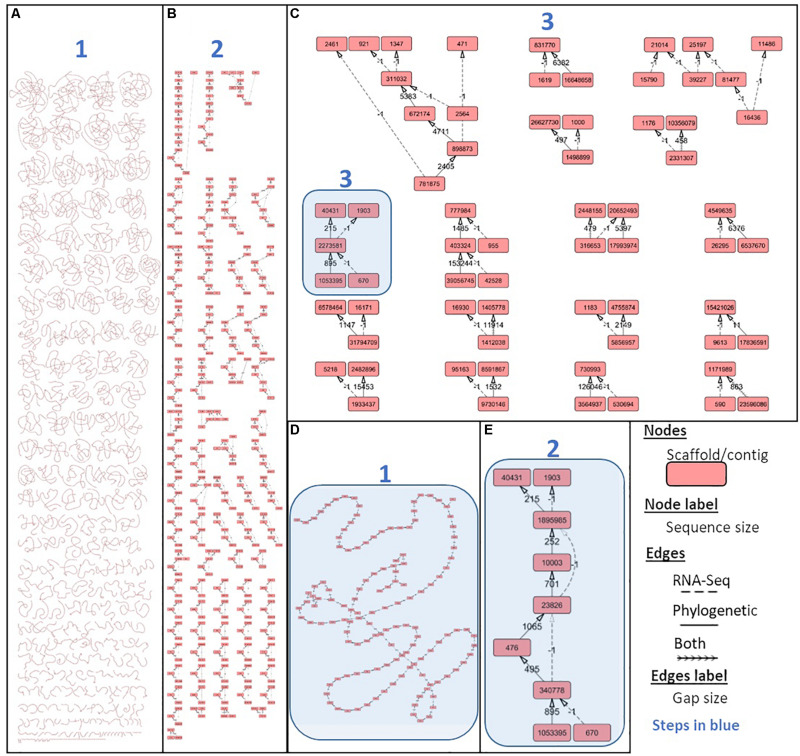
Integrating phylogenetic and gene-based secondary scaffolding with Scaff2links. Scaff2link allows the import of linkages established by both AGOUTI for gene-based scaffolding and Ragout for reference-assisted scaffolding (see section “Materials and Methods”). The results of the process of conservative union from (1) the initial state with links parsed from Ragout and AGOUTI to (2) the linear chain simplification and (3) the directed acyclic graph simplification is respectively shown on **(A)**, **(B)**, and **(C)** were connected components resolved up to a single node are no longer displayed. One connected component at the initial step 1, 2, and 3 are respectively shown on **(D)**, **(E)**, and **(C)** and highlighted in blue. Graph structure and different steps (1–3) are detailed in Supplementary Material.

The Scaff2link steps presented on the general workflow (Figure 2) combine different pieces of evidence allowing to avoid errors at secondary scaffolding steps (see section “Materials and Methods”). Scaff2link allows conservative integration of both linkage information built by AGOUTI and Ragout. When importing links from AGOUTI based on RNA-Seq data, the minimum number of reads to consider a linkage is an option; this option has been tested with 10 and 100 minimum RNA-Seq reads leading to Ma_sr-lr_union10 and Ma_sr-lr_union100 assemblies, respectively. A general picture of the assembly states during scaff2link processing is shown in Figure 6, with intermediary steps presented on Figures 6A,B and the final step depicted in Figure 6C, corresponding to Ma_sr-lr_union100. In this final step, there were still unresolved links due to contradictory information (potentially linked with scaffolding errors) or fork-like graph structures that are impossible to resolve without introducing potential misassembles. Then, the quality has been assessed with the same metrics as those used before.

First, NG50 values of reference-assisted scaffolding are high with 17 and 19 Mb (Figure 3), corresponding to Ma_sr-lr_union10 and Ma_sr-lr_union100, respectively. These values are close to Ma_sr-lr_phylo (20 Mb) and still higher than *P. alecto* (16 Mb).

Second, in terms of quality for gene annotation (Figure 4A), results for scaff2link assemblies are very similar to the reference-assisted assembly Ma_sr-lr_phylo. Interestingly, for Ma_sr-lr_union100 compared to Ma_sr-lr_phylo, one more BUSCO annotation is complete (from fragmented) and presents as a single copy, indicating a slightly better scaffolding quality.

Third, regarding DNA-Seq read mapping (Figure 4B), results are again very similar to Ma_sr-lr_phylo. More detailed changes in read counts are given in Table 1. There are more reads mapped on Ma_sr-lr_union100 assembly than on Ma_sr-lr_phylo assembly (+20 reads), there are even more reads mapped properly in pairs (+358). Being slightly better than other assemblies, the Ma_sr-lr_union100 was chosen to perform annotation and the identification of repeats, and for the final upload in ENA.

**TABLE 1:** Details for DNA-Seq consistency for Scaff2links assemblies in comparison to reference-assisted scaffolding.

Features*	Scaffolds «Phylo»	Difference with	
		
		«union100»	«union10»
Number of mapped^#^	764,982,145	+20	−126
Number of properly paired	758,804,408	+358	+26
Number of Singletons	2,288,435	−26	+94
Number with mate mapped to a different fragment	309,158	−132	+276
Number with mate mapped to a different fragment (mapQ ≥ 5)	219,998	+16	+48

*More detailed view of DNA-Seq consistency within scaffold assemblies after secondary scaffolding. The table focus on differences between union100 and union10 assemblies which differ in the minimum number of reads to support a linkage through mRNA-Seq. DNA-Seq consistency is expressed in terms of short-read alignment statistics. ^#^“Mapped” stands for the number of reads mapped. “Properly paired” stands for the number of reads that are properly paired (right strand and insert size range). A Singleton is a read with an unmapped mate. mapQ corresponds to a pared quality score of a read alignment.

### Genome Annotation

A total of 19,823 coding genes were annotated on Ma_sr-lr_union100 scaffold assembly, as shown in Table 2 and compared to 19,260 for *P. vampyrus* annotation (PteVam2/RefSeq 101). Therefore, in terms of annotated gene count, both are similar (19.8 k vs 19.3 k). More mRNAs were annotated for *P. medius* (42 k vs 33 k), this type of difference being expected when different annotation methods are used. Finally, as shown in Table 3, genes including introns are covering 40.3% of the genome and CDS only (coding sequence) 3.7%. Although the majority of CDSs are complete, some (5 k out of 39 k) miss either a start codon and/or a stop codon. This would require manual inspection for each CDS to elucidate the cause of incompleteness; each non-complete CDS might be the result of the gap presence (bench of Ns), more problematically a mis-assembly or it may be an event of pseudogenization. Using RepeatMasker, 22.5% of the genome was identified as already known interspersed repeats, discovered and annotated in another species and present in the repeat database (repbase), as shown Table 4.

**TABLE 2:** Gene annotation statistics by features, summarized for *P. medius* and *P. vampyrus* genome annotations, considering only protein coding genes.

Features	Number		Total length		Shortest length		Longest length		Mean	
					
	*P. medius*	*P. vampyrus*	*P. medius*	*P. vampyrus*	*P. medius*	*P. vampyrus*	*P. medius*	*P. vampyrus*	*P. medius*	*P. vampyrus*
Gene	19,823	19,260	799,771,699	821,869,377	35	132	1,646,990	2,090,425	40,346	42,672
mRNA	42,119	33,311	2,268,593,933	1,611,530,784	35	132	1,646,990	2,090,425	53,862	48,378
Exons	502,353	372,960	130,167,394	94,948,423	1	1	17,601	17,106	259	255
Introns	460,234	339,649	2,139,347,007	1,517,261,659	1	1	863,371	923,485	4,648	4,467
CDS	39,106	33,311	73,655,104	59,627,155	46	96	102,629	102,513	1,883	1,790

**TABLE 3:** Coverage of genome annotation.

Statistics*	Value
% of genome covered by genes	40.3
% of genome covered by CDS	3.7
Mean mRNAs per gene	2
Mean exons per mRNA	12
Mean introns per mRNA	11
Overlapping genes	3,344
Contained genes	1,123
CDS: complete	37,076
CDS: start, no stop	684
CDS: stop, no start	1,256
CDS: no stop, no start	3,103

*The table summarizes statistics representing the coverage of the genome annotation for protein-coding genes only, produced with GAG (Genome Annotation Toolkit) software (https://github.com/genomeannotation/GAG).

**TABLE 4:** Interspersed repeats annotated in *P. medius* genome sequence using RepeatMasker.

Class*	Sub-class	Count	Cumulative size (bp)	Percentage in the genome (%)
DNA	–	1,010	191,112	0.01
	PiggyBac	1,954	721,278	0.04
	TcMar-Mariner	648	125,351	0.01
	TcMar-Tc2	1,737	618,278	0.03
	TcMar-Tigger	32,925	11,660,016	0.59
	hAT	824	208,125	0.01
	hAT-Ac	478	217,854	0.01
	hAT-Blackjack	3,968	1,037,449	0.05
	hAT-Charlie	72,918	17,479,065	0.89
	hAT-Tag1	403	144,892	0.01
	hAT-Tip100	12,415	4,240,959	0.22
LINE	CR1	3,663	1,159,469	0.06
	L1	399,046	293,952,163	14.97
	L2	53,043	19,005,727	0.97
	RTE-BovB	1,110	618,176	0.03
	RTE-X	1,646	676,407	0.03
LTR	–	1,631	574,128	0.03
	ERV1	44,596	23,334,633	1.19
	ERVK	5,915	1,601,327	0.08
	ERVL	41,775	22,884,779	1.17
	ERVL-MaLR	75,722	27,922,114	1.42
	Gypsy	1,965	693,212	0.04
RC	Helitron	332	113,660	0.01
SINE	5S	11,447	2,005,503	0.10
	MIR	67,536	9,536,267	0.49
	tRNA	1,887	252,641	0.01
	tRNA-5S	2,413	393,491	0.02
Unknown	–	639	130,749	0.01
Total	Interspersed	844,981	441,707,409	22.50

*Results from RepeatMasker are summarized by repeat class and the repeat class below 0.01% is masked but was taken into account for the total counts.

### Phylogenetic Analysis

**FIGURE 7:**
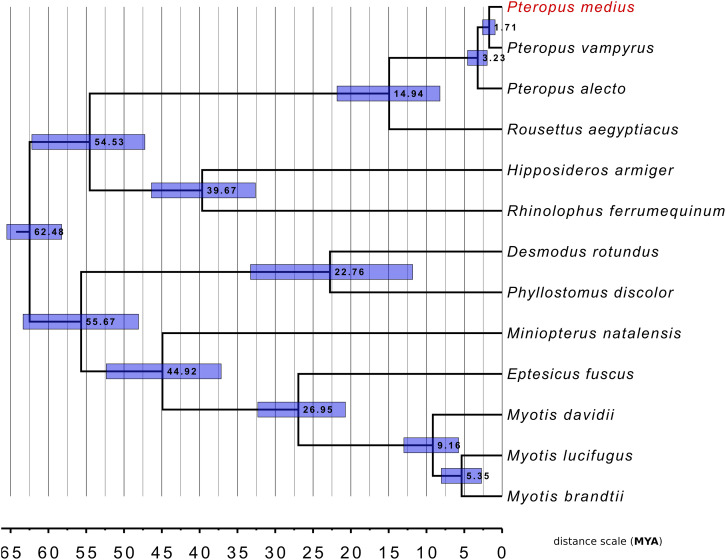
Phylogenetic classification of *P. medius* among other bats and within *Pteropus* species. The distance scale unit is MYA (Million Years Ago). All bootstrap values were 100%. Horizontal blue bars around each node represents the 95% confidence interval relative to this node age.

To investigate genetic relationships between *P. medius* and the other bat species for which the complete genome sequences are available, we performed the phylogenetic analysis with 12 published bat genomes with RefSeq proteomes available (Figure 7). A total of 9,710 SCO were found thanks to OrthoFinder. Following multiple sequence alignment and post-alignment analysis (described in the section “Materials and Methods”), the concatenated MSA contained 3,694,685 positions of which 473,736 were variable in at least one species accounting for 94,420 distinct alignment patterns. All bootstrap analyses (100%) were consistent with the topology of this tree. This analysis strongly supports that *P. medius* is phylogenetically closer to *P. vampyrus* than to *P. alecto*. Our timescale, based on protein alignments, indicates a divergence time of 1.57 MY (0.9–2.57948) between both *P. medius* and *P. vampyrus*, and 2.98 MY (1.97795–4.56922) between them and *P. alecto*. Finally, as angiotensin converting enzyme 2 (ACE2) is considered to be the entry receptor for SARS-CoV-2, the virus responsible for the current pandemic of COVID-19 (Zhou et al., 2020), we compared the sequences of ACE2 (805 amino acids) between *P. medius* and two *Rhinolophus* bat species, *R. ferrumequinum* and *R. sinicus*, thought to be a natural reservoir of coronaviruses (Ge et al., 2013). We observed 82.1% of identity of *P. medius* with both *Rhinolophus* bat species. The percentage of similitude (based on BLOSSUM62 matrix) of *P. medius* sequence is respectively of 90.3 and 89.8% for *R. ferrumequinum* and *R. sinicus* (Supplementary Figure S4). Of note, the percentage of similitude between *R. ferrumequinum* and *R. sinicus* is 95.3%.

## Discussion

In this study, we sequenced and assembled a solid scaffold for the *P. medius* genome. We obtained NG50 of 353 kb, on which we were able to annotate most of the genes as shown by BUSCO analysis. In addition, we report here that the process of secondary scaffolding is beneficial in terms of assembly quality (6.1x of Nanopore long-reads and 57.4x Illumina paired-end) with sequencing cost that remains relatively moderate, making thus this approach rather attractive. Although it would probably be insufficient to provide a resourceful assembly if closely related species have not been fully sequenced, we show in this study that it is possible to take advantages of published genomes from close species to provide a good quality assembly. In a context where international consortiums provide the scientific community with high quality genome sequences at chromosome level (Genome 10K Community of Scientists, 2009; Teeling et al., 2018), for a genus without available genome sequences, there will be more resources available for reference-assisted scaffolding methods and for sequencing species of interest at lower cost.

Our results suggest that the combination of different sources of information for secondary scaffolding is necessary to limit the introduction of misassembles and improve quality; we thus developed the scaff2link software, which is compatible and reusable with Ragout and AGOUTI. We have shown that the Ma_sr-lr_union100, produced by scaff2link, is slightly better than other assemblies. Indeed, Scaff2links prevents the linkage of fragments within a new scaffold if contradictory information exists between gene-based and synteny-based secondary scaffolding. This is expected to limit the number of scaffolds produced with mis-assemblies. Finally, our results suggest the advantage of the utilization of the Ma_sr-lr_union100 assembly for gene annotation and subsequent analysis of regulatory features including NGS applications, such as RNA-Seq. Ma_sr-lr_union100 assembly could also be used to perform genome-wide comparative studies to identify or compare loci, including coding sequences or regulatory motifs. However, the fact that some contiguities in the genome are based on prediction (from secondary scaffolding), rather than on actual data might be problematic for some analysis. As example, for use as a reference genome for Ragout, it would be preferable to use Ma_sr-lr assembly.

Similarly to the previously sequenced *E. spelaea* bat genome (Wen et al., 2018), the main class of repeats present in *P. medius* genome are LINE (long-interspersed nuclear elements), covering ∼16% of the genome. However, the percentage of total genome coverage for interspersed repeats found in this study was lower than previous reports about closely related bat species (Wen et al., 2018). This difference is linked with the lower sensitivity of the tool used to search similarities between the genome and the repeat database (RMBlast, used in this study). To our current knowledge there is no published work with significant statistical support allowing the resolution of phylogenetic relationship of *P. medius* with *P. alecto* and *P. vampyrus*. Phylogenetic analysis shown the clustering of *P. medius* genome with two other *Pteropus* bat species, *P. alecto* and *P. vampyrus*. The overall tree topology for bats is concordant with other studies (Teeling et al., 2005; Hawkins et al., 2019). Finally, the tree topology is coherent with the geographical distribution of those bats: *P. medius* (Molur et al., 2008) and *P. vampyrus* (Bates et al., 2008), which clustered together, are both present on the Asian continent. On the other hand, phylogenetically more distant species *P. alecto* is mainly located in Australia (Roberts et al., 2017). We estimated in this study the divergence time between *Pteropus* species lower than the minimum ranges reported on Time Tree database. However, some other studies reported divergence times focused on *Pteropus* genus even lower than ours (Almeida et al., 2014); in that study the 95% confidence interval for the divergence time between *P. vampyrus* and *P. medius* (0.5–1.4) overlaps with ours (0.9–2.57948). We conducted our timescale estimation using SCO sequences, where the proportion of site under non-neutral selection may vary among branches. We acknowledge that time divergence estimations on branch with a greater proportion of sites under positive selection might be estimated higher than the real divergence time; inversely time divergence estimations on branches with a higher proportion of sites under negative selection might be estimated lower.

Indian *P. medius* bats have been suggested to host coronaviruses (Anthony et al., 2013; Yadav et al., 2020). To underline the importance of having available an annotated *P. medius* genome, we analyzed the ACE2 gene which is considered to code for the entry receptor for coronavirus SARS-CoV-2, responsible for the current pandemic of COVID-19 (Zhou et al., 2020). Comparison of the sequences of ACE2 between *P. medius* and *R. sinicus*, thought to be a natural reservoir of coronaviruses (Ge et al., 2013) revealed 82.1% of identity and 89.8–90.3% of similarity (Supplementary Figure S3). Although this is a preliminary result, it emphasizes the interest of having ready-to-use genomic resources for a maximum of species, for the further in-depth analysis.

Altogether, these results confirm that a lower depth of sequencing is enough to obtain reliable genome sequence using secondary scaffolding approaches and demonstrate the benefits of the scaff2link application, described in this article. The genome sequence is now available to the scientific. In addition, as bats display many unique biological features among mammals (Racey, 2015), growing number of new bat species are expected to be sequenced, some of them within the Bat 1K project for sequencing all bats’ species (Teeling et al., 2018). A meta-analysis of bat phylogenetic and positive selection recently suggested a number of genes known to be primarily related to immune responses (Hawkins et al., 2019) and their further functional analysis will allow the understanding of their role in hosting different viruses by bats. Increasing the availability of different bat genomes is indeed essential for a better understanding of the genetic and evolutionary mechanisms that underlie the adaptations specifically to bats, such as their ability to fly which is unique among mammals, their metabolic adaptation, as well as the immuno-virological peculiarities associated to their capacity to both host and transmit Nipah virus as well as the other viruses highly pathogenic to humans.

## Data Availability Statement

The datasets generated for this study can be found in the European Nucleotide Archive (ENA), N° PRJEB32728, ERP115442. Some files important for the reproducibility of this work are available at: https://github.com/jfouret/pMed_genomeData.

## Ethics Statement

Ethical approval was not required for this study according to local regulations as tests were performed in the course of regular health checks by veterinarians of the Vienna Zoo.

## Author Contributions

JF, BH, CL-L, and MB-B conceived, designed, and supervised the study. DP performed the sampling of biological material. JF, NA, and FE performed the experimental processing of the samples. SC and MG performed, respectively, MinION and Illumina sequencing with inputs from JF for MinION sequencing. JF led the software development and performed the sequence analysis, genome assembly, and gene annotation. MB contributed significantly for genome assembly, evaluation, and genome annotation. AG, JF, and JL brought methodological inputs. FB, JF, and J-NV performed the phylogenic analysis. JF wrote the initial manuscript. FB, AG, BH, CL-L, and JL did the proofreading. All authors read and approved the final manuscript.

## Conflict of Interest

JF, MB, MG, AG, and CL-L were employed by the company ViroScan3D. The remaining authors declare that the research was conducted in the absence of any commercial or financial relationships that could be construed as a potential conflict of interest.

## Footnotes

1 docker://davidemms/orthofinder sha256:fbd408ae21ce

2 https://paleobiodb.org

3 http://www.timetree.org
