# Supplementary material for: Sequencing the Genome of Indian Flying Fox, Natural Reservoir of Nipah Virus, Using Hybrid Assembly and Conservative Secondary Scaffolding
Source: Front Microbiol. 2020 Jul 29;11:1807. doi: 10.3389/fmicb.2020.01807 (PMC7403528; doi:10.3389/fmicb.2020.01807)
Supplement: Supplementary file 1 [file Data_Sheet_1.docx]

# SUPPEMENTARY DATA:

**Sequencing of the genome of Indian Flying Fox*,* natural reservoir of Nipah virus, using hybrid assembly and conservative secondary scaffolding**

Julien Fouret^1,2^, Frédéric G. Brunet^3^, Martin Binet^1,2^, Noémie Aurine^1^, Francois Enchéry^1^, Séverine Croze^7^, Marie Guinier^2^, Abdelghafar Goumaidi^2^, Doris Preininger^6^, Jean-Nicolas Volff^3^, Marc Bailly-Bechet^8^, Joël Lachuer^5,7^, Branka Horvat^1^*, Catherine Legras-Lachuer^2,4^*

^1^ International Center for Infectiology Research (CIRI), INSERM U1111, CNRS UMR5308, Université de Lyon, Université Claude Bernard Lyon 1, Ecole Normale Superieure de Lyon, Lyon, France

^2^ Viroscan3D, Trévoux, France

^3^ Institut de Génomique Fonctionnelle de Lyon, Université de Lyon, CNRS UMR 5242, Ecole Normale Supérieure de Lyon, Université Claude Bernard Lyon 1, Lyon, France.

^4^ Ecologie Microbienne, CNRS UMR5557, Université Lyon 1, LEM, CNRS, INRA, VetAgro Sup, France

^5^ Cancer Research Center of Lyon, INSERM 1052/CNRS 5286, Université de Lyon, France

^6^ Tiergarten Schönbrunn, Vienna, Austria

^7^ Plateforme Profilexpert, Université Claude Bernard Lyon 1

^8^ Université Nice Sophia Antipolis, INRA, CNRS, ISA, France

**Running tittle**: Hybrid assembly of *Pteropus medius* genome

* Equal contribution

^#^ Corresponding authors: [julien@fouret.me](mailto:julien@fouret.me) [catherine.lachuer@univ-lyon1.fr](mailto:catherine.lachuer@univ-lyon1.fr) [branka.horvat@inserm.fr](mailto:branka.horvat@inserm.fr)

# SUPPEMENTARY FIGURES


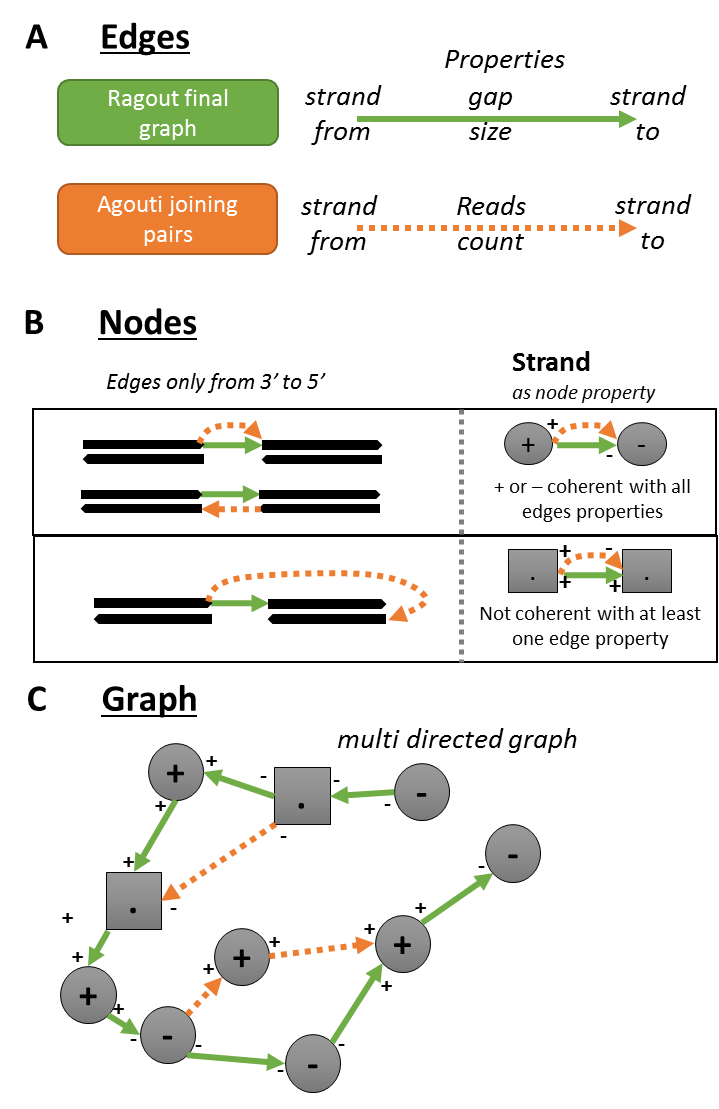


**Figure S1: Graph structure in scaff2links.** (A) Edges are directed from one scaffold to another. Strands of both scaffolds based on the sequence kept in memory are properties of the nodes. When an edge is added, its direction can be inverted to match the strand property from the nodes. (B) A node, representing a scaffold/contig, is initiated with no strand value. When an edge is added, connected nodes are taking the strand property to be consistent with the edge. If, even by inverting the edge, there is no strand consistency, the node is marked as inconsistent (square shape). (C) This final graph presents a multi-directed graph, each node representing a specific strand of a scaffold, except inconsistent nodes that require special care.


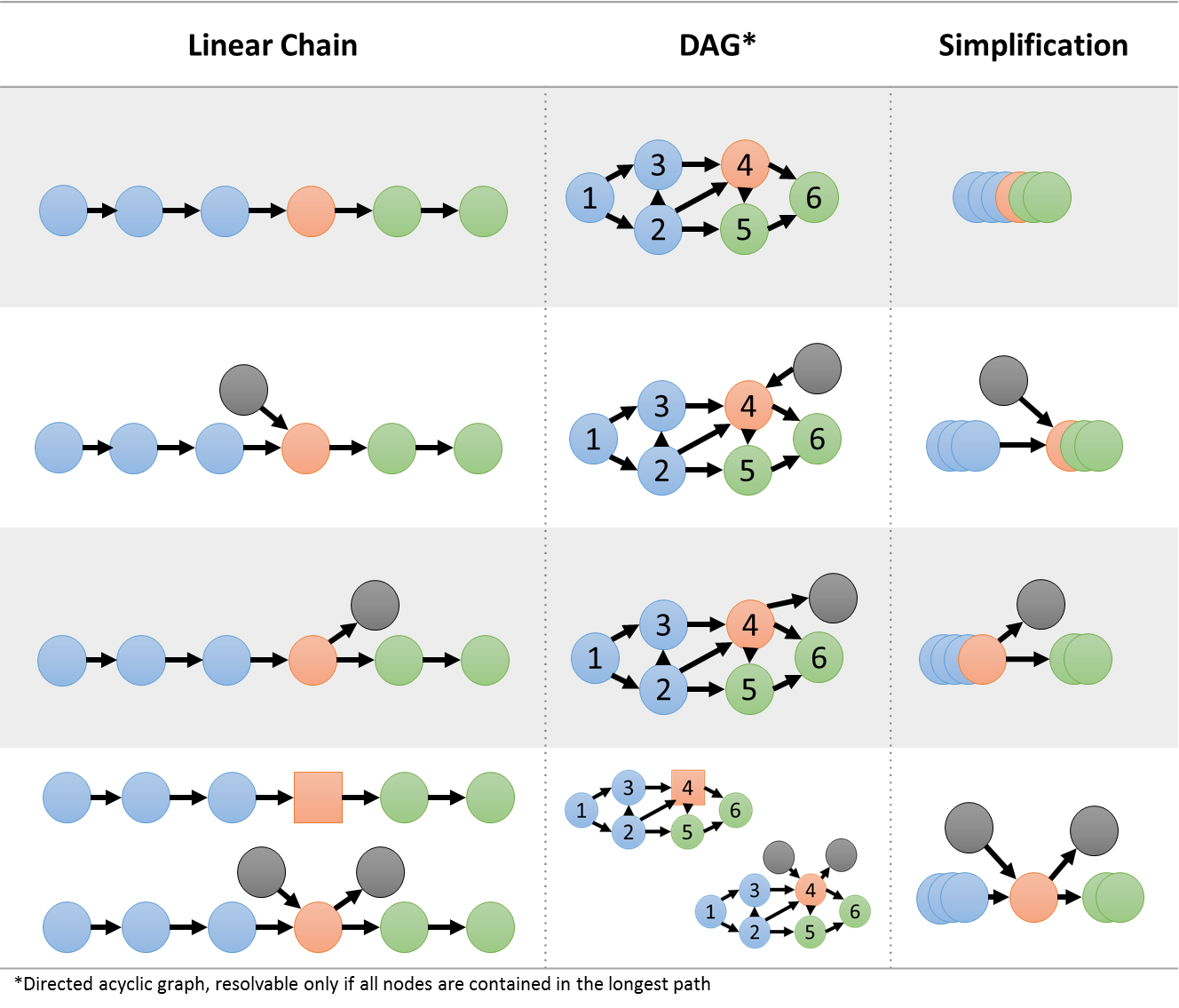


**Figure S2: Principles implemented in scaff2links.** This figure represents different case of simplification for either linear chain or directed acyclic graph. The square shapes represent nodes with aberrant strand orientation (see graph structure in scaff2link in Figure 1S).

#
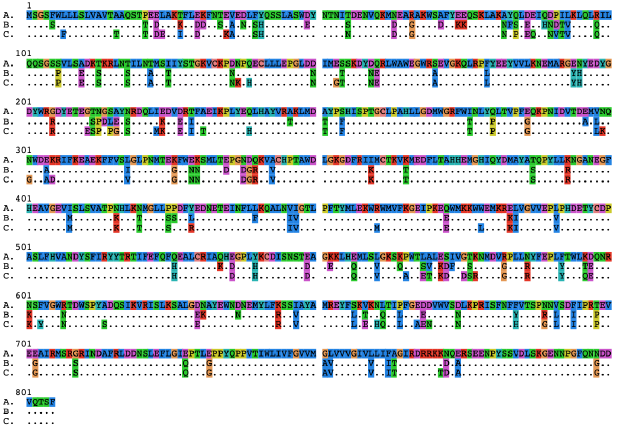


**Figure S3:** **Multiple alignment of ACE2 protein homologs**. Sequence A: *Pteropus medius*(from assembly); Sequence B: *Rhinolophus ferrumequinum* E2DHI2; Sequence C: *Rhinolophus sinicus* U5WHY8 for ACE2.

**
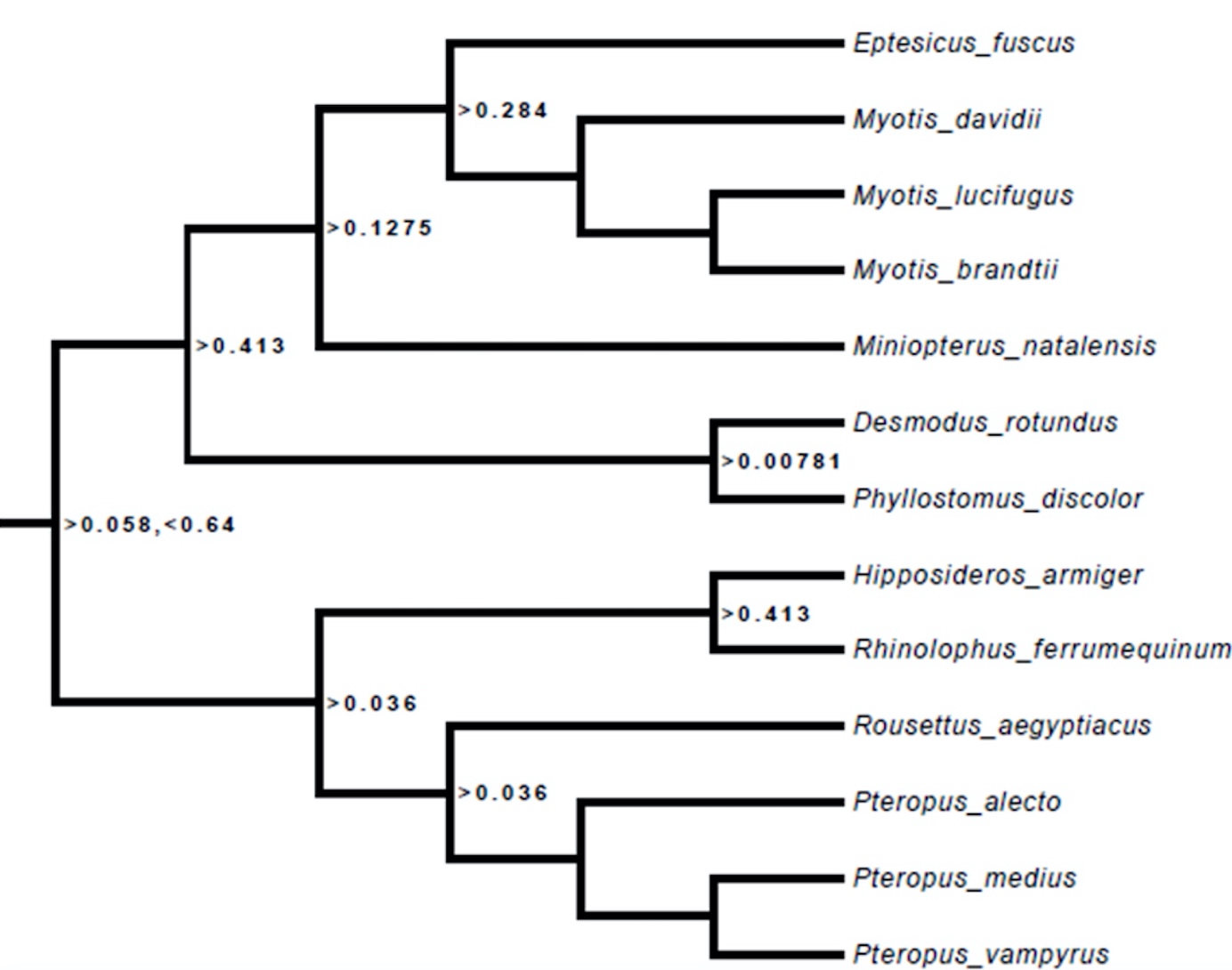
**

**Figure S4: Constraints for mcmctree applied for molecular timescale estimation.** Units are in 100 MYA (Million Years Ago). All constraints came from fossil data as explained in the material and method section, except for ‘<0.64’ for rootAge, that is taken from the 95% confidence interval of Chiroptera age on Time Tree database.

# SUPPLEMENTARY TABLE

**Table 1S:** An overview of different assemblies of *P. giganteus* compared to available assemblies for *P. alecto* and *P. vampyrus*.

| Features* | Ma_sr-lr | Ma_sr-lr_phylo | Ma_sr-lr_rna-seq | Ma_sr-lr_union10 | Ma_sr-lr_union100 | pteAle1 | pteVam2 |
| --- | --- | --- | --- | --- | --- | --- | --- |
| Number of scaffolds  (>= 0 bp) | 29,459 | 16,119 | 27,397 | 16,171 | 16,113 | 65,598 | 36,094 |
| Number of scaffolds  (>= 50000 bp) | 7,360 | 442 | 6,287 | 548 | 459 | 471 | 986 |
| Total length (Mb)  (>= 0 bp) | 1,968 | 1,986 | 1,970 | 1,985 | 1,985 | 1,986 | 2,198 |
| Total length (Mb)  (>= 50000 bp) | 1,820 | 1,947 | 1,842 | 1,947 | 1,947 | 1,948 | 2,065 |
| Largest contig (Mb) | 3 | 68 | 3 | 45 | 68 | 70 | 43 |
| GC (%) | 39.82 | 39.2 | 39.82 | 39.82 | 39.82 | 39.71 | 39.81 |
| N50 (kb) | 347 | 19,890 | 466 | 16,705 | 18,871 | 16,440 | 5,954 |
| N75 (kb) | 159 | 7,787 | 197 | 5,795 | 7,402 | 6,841 | 2,257 |
| L50 | 1,574 | 30 | 1,182 | 39 | 32 | 35 | 102 |
| L75 | 3,658 | 67 | 2,803 | 92 | 73 | 81 | 255 |
| Number N's per 100 kbp | 55.66 | 919.23 | 160.38 | 900.63 | 903.24 | 2094.25 | 8235.54 |

*GC (%) stands for the percentage of G and C in the genome sequence. N50 and N75 are respectively defined as a scaffold size, such as 50% and 75% of the genome sequence, included in scaffold larger than N50 and N75 values. L50 and L75 are defined as the minimum number of scaffolds whose size sum reach respectively 50% and 75% of total genome size. The presence of ‘N’ in assembly sequence might be a residual ambiguity from sequencing but is most probably part of batch of ‘N’s inserted at the scaffolding step based on an estimation of the gap size for each linkage.

# SUPPLEMENTARY METHODS

## Setting up the long read assembly

Assembly tests were performed with illumina DNA-Seq data using ABySS (v1.9.0) (Simpson et al., 2009), SOAPdenovo (v 2.04) (Li et al., 2009), and spades (v3.11.0) (Antipov et al., 2016; Bankevich et al., 2012) however the max NG50 reached was 24kb only, of note scaffolding procedure based on illumine paired-end library, either integrated within assemblers or using SSPACE did not improve the results.

We then tried hybrid methods for assembly using hybrid function of SPAdes. The best parameters at the previous step were re-used. Indeed, given the high computational cost of hybrid-based methods, testing with different values of k was not conceivable. Unfortunately, during the step in which long reads are aligned to the De Bruijn graph, less than 50 out of 50000 long reads were reported in early steps. Despite the advice to use uncorrected reads in SPAdes manual, we tried to correct the reads. It has been tested using LoRDEC (v0.8) (Salmela and Rivals, 2014) and JaBbA (github.com/biointec/jabba a2fa477) (Miclotte et al., 2016). LoRDEC correction algorithm led to almost no long read actually corrected. We try to run JaBbA algorithm but had to stop it after 20,000 hours CPU time of computation (32 threads spread on 4 Intel(R) Xeon(R) CPU E5-4627 v2 @ 3.30GHz and 1.5TB Ram spread on 48 modules of 32GB DDR3-1866 LRDIMM PC3-14900L). Finally, a hybrid assembly was produced by running MaSuRCA. It took 314 hours real time to complete with 16 threads on the same computer.

## Algorithm of scaff2links

Schematic presentation of the graph construction is specified on **Figure** 2. Phylogenetic links are parsed from ‘.*_scaffolds.agp’ (Ragout output) and gene-based links are parsed from ‘.*join_pairs.noise_free.txt’ (AGOUTI output). Linear paths are simplified when possible (**Figure** 3). The following pseudocode is used to find directed acyclic graph (DAG) pattern that can be simplified:

Take graph **G**

Initiate an empty set **S** of paths

Copy **G** to **G’**

Remove bridges from graph **G’**

For each connected component **c** in **G’**:

If **c** is a DAG:

Calculate the longest path **P** in **c**

If all nodes from **c** are included in **P**:

Add **P** to **S**

Then **S** contains longest path from all DAG that can be simplified. The networkx python package is used for basic graph functions. DAG pattern is then resolved as specified on Figure **S3**.

## Data linked with Figure 3B

As described the graphic from Figure 3B were produced using outputs from `samtools flastats`. Below are given the complete outputs from this software for each assembly.

- **Ma_sr-lr :**

772211240 + 0 in total (QC-passed reads + QC-failed reads)

0 + 0 secondary

0 + 0 supplementary

0 + 0 duplicates

764929546 + 0 mapped (99.06% : N/A)

772211240 + 0 paired in sequencing

386105620 + 0 read1

386105620 + 0 read2

758733998 + 0 properly paired (98.25% : N/A)

762630240 + 0 with itself and mate mapped

2299306 + 0 singletons (0.30% : N/A)

325454 + 0 with mate mapped to a different chr

228584 + 0 with mate mapped to a different chr (mapQ>=5)

- **Ma_sr-lr_phylo :**

772211240 + 0 in total (QC-passed reads + QC-failed reads)

0 + 0 secondary

0 + 0 supplementary

0 + 0 duplicates

764982145 + 0 mapped (99.06% : N/A)

772211240 + 0 paired in sequencing

386105620 + 0 read1

386105620 + 0 read2

758804408 + 0 properly paired (98.26% : N/A)

762693710 + 0 with itself and mate mapped

2288435 + 0 singletons (0.30% : N/A)

309158 + 0 with mate mapped to a different chr

219998 + 0 with mate mapped to a different chr (mapQ>=5)

- **Ma_sr-lr_rna :**

772211240 + 0 in total (QC-passed reads + QC-failed reads)

0 + 0 secondary

0 + 0 supplementary

0 + 0 duplicates

764973877 + 0 mapped (99.06% : N/A)

772211240 + 0 paired in sequencing

386105620 + 0 read1

386105620 + 0 read2

758784462 + 0 properly paired (98.26% : N/A)

762679722 + 0 with itself and mate mapped

2294155 + 0 singletons (0.30% : N/A)

323024 + 0 with mate mapped to a different chr

227284 + 0 with mate mapped to a different chr (mapQ>=5)

- **Ma_sr-lr_union10:**

772211240 + 0 in total (QC-passed reads + QC-failed reads)

0 + 0 secondary

0 + 0 supplementary

0 + 0 duplicates

764982019 + 0 mapped (99.06% : N/A)

772211240 + 0 paired in sequencing

386105620 + 0 read1

386105620 + 0 read2

758804434 + 0 properly paired (98.26% : N/A)

762693490 + 0 with itself and mate mapped

2288529 + 0 singletons (0.30% : N/A)

309434 + 0 with mate mapped to a different chr

220483 + 0 with mate mapped to a different chr (mapQ>=5)

- **Ma_sr-lr_union100:**

772211240 + 0 in total (QC-passed reads + QC-failed reads)

0 + 0 secondary

0 + 0 supplementary

0 + 0 duplicates

764982165 + 0 mapped (99.06% : N/A)

772211240 + 0 paired in sequencing

386105620 + 0 read1

386105620 + 0 read2

758804766 + 0 properly paired (98.26% : N/A)

762693756 + 0 with itself and mate mapped

2288409 + 0 singletons (0.30% : N/A)

309026 + 0 with mate mapped to a different chr

220014 + 0 with mate mapped to a different chr (mapQ>=5)

# PAML CONFIGURATION FOR MCMCTREE

- Step 1 in dir1

/dir1$ more mcmctree.ctl

seed = -1

seqfile = ../aln_500k.fa

treefile = ../tree_calib.nw

mcmcfile = mcmc.txt

outfile = out.txt

seqtype = 2

usedata = 3

clock = 2

RootAge = 'B(0.558, 0.64, 0.025, 0.05)'

model = 2

aaRatefile = ../paml4.9j/dat/lg.dat

alpha = 0.5

ncatG = 5

cleandata = 0

BDparas = 1 1 0.1

kappa_gamma = 6 2

alpha_gamma = 1 1

rgene_gamma = 2 20 1

sigma2_gamma = 1 10 1

print = 1

burnin = 2000

sampfreq = 10

nsample = 20000

- Step 2 in dir2: (after copying the out.BV file from dir1 to dir2)

/dir2$ more mcmctree.ctl

seed = -1

seqfile = ../aln_500k.fa

treefile = ../tree_calib.nw

mcmcfile = mcmc.txt

outfile = out.txt

seqtype = 2

usedata = 2 in.BV

clock = 2

RootAge = 'B(0.558, 0.64, 0.025, 0.05)'

model = 2

aaRatefile = ../paml4.9j/dat/lg.dat

alpha = 0.5

ncatG = 5

cleandata = 1

BDparas = 2 2 .1

kappa_gamma = 6 2

alpha_gamma = 1 1

rgene_gamma = 2 20 1

sigma2_gamma = 1 10 1

print = 1

burnin = 4000

sampfreq = 10

nsample = 25000

# SUPPLEMENTARY FILE

Supplementary files are available at https://github.com/jfouret/pMed_genomeData

**annotation.gff3.gz :**

This file contains the gene annotation that has been described in this article. It uses the widely used GFF3 text format (see https://www.ensembl.org/info/website/upload/gff3.html).

**aln.fa.bz2 :**

This file contains the single copy orthologs concatenated after multiple sequence alignment and filtering.

**assembly files :**

All assemblies that are not on ncbi are available in this repository

# SUPPLEMENTARY BIBLIOGRAPHY

Antipov, D., Korobeynikov, A., McLean, J.S., and Pevzner, P.A. (2016). hybridSPAdes: an algorithm for hybrid assembly of short and long reads. Bioinformatics *32*, 1009–1015.

Bankevich, A., Nurk, S., Antipov, D., Gurevich, A.A., Dvorkin, M., Kulikov, A.S., Lesin, V.M., Nikolenko, S.I., Pham, S., Prjibelski, A.D., et al. (2012). SPAdes: A New Genome Assembly Algorithm and Its Applications to Single-Cell Sequencing. J. Comput. Biol. *19*, 455–477.

Li, R., Yu, C., Li, Y., Lam, T.W., Yiu, S.M., Kristiansen, K., and Wang, J. (2009). SOAP2: An improved ultrafast tool for short read alignment. Bioinformatics *25*, 1966–1967.

Miclotte, G., Heydari, M., Demeester, P., Rombauts, S., Van de Peer, Y., Audenaert, P., and Fostier, J. (2016). Jabba: hybrid error correction for long sequencing reads. Algorithms Mol. Biol. *11*.

Salmela, L., and Rivals, E. (2014). LoRDEC: accurate and efficient long read error correction. Bioinformatics *30*, 3506–3514.

Simpson, J.T., Wong, K., Jackman, S.D., Schein, J.E., and Jones, S.J.M. (2009). ABySS: A parallel assembler for short read sequence data. 1117–1123.
